# miR-6807-5p Inhibited the Odontogenic Differentiation of Human Dental Pulp Stem Cells Through Directly Targeting METTL7A

**DOI:** 10.3389/fcell.2021.759192

**Published:** 2021-11-01

**Authors:** Ning Wang, Xiao Han, Haoqing Yang, Dengsheng Xia, Zhipeng Fan

**Affiliations:** ^1^Laboratory of Molecular Signaling and Stem Cells Therapy, Beijing Key Laboratory of Tooth Regeneration and Function Reconstruction, Capital Medical University School of Stomatology, Beijing, China; ^2^Department of General Dentistry and Integrated Emergency Dental Care, Capital Medical University School of Stomatology, Beijing, China; ^3^Research Unit of Tooth Development and Regeneration, Chinese Academy of Medical Sciences, Beijing, China

**Keywords:** miR-6807-5p, human dental pulp stem cells (DPSCs), METTL7A, odontogenic differentiation, SNRNP200, mineralized induction

## Abstract

**Background:** Tooth tissue regeneration mediated by mesenchymal stem cells (MSCs) has become the most ideal treatment. Although the known regulatory mechanism and some achievements have been discovered, directional differentiation cannot effectively induce regeneration of tooth tissue. In this study, we intended to explore the function and mechanism of miR-6807-5p and its target gene METTL7A in odontogenic differentiation.

**Methods:** In this study, human dental pulp stem cells (DPSCs) were used. Alkaline phosphatase (ALP), Alizarin red staining (ARS), and calcium ion quantification were used to detect the odontogenic differentiation of miR-6807-5p and METTL7A. Real-time RT-PCR, western blot, dual-luciferase reporter assay, and pull-down assay with biotinylated miRNA were used to confirm that METTL7A was the downstream gene of miR-6807-5p. Protein mass spectrometry and co-immunoprecipitation (Co-IP) were used to detect that SNRNP200 was the co-binding protein of METTL7A.

**Results:** After mineralized induction, the odontogenic differentiation was enhanced in the miR-6807-5p-knockdown group and weakened in the miR-6807-5p-overexpressed group compared with the control group. METTL7A was the downstream target of miR-6807-5p. After mineralized induction, the odontogenic differentiation was weakened in the METTL7A-knockdown group and enhanced in the METTL7A-overexpressed group compared with the control group. SNRNP200 was the co-binding protein of METTL7A. The knockdown of SNRNP200 inhibited the odontogenic differentiation of DPSCs.

**Conclusion:** This study verified that miR-6807-5p inhibited the odontogenic differentiation of DPSCs. The binding site of miR-6807-5p was the 3′UTR region of METTL7A, which was silenced by miR-6807-5p. METTL7A promoted the odontogenic differentiation of DPSCs. SNRNP200, a co-binding protein of METTL7A, promoted the odontogenic differentiation of DPSCs.

## Introduction

Tooth loss is a common and frequently occurring disease in humans. The function of mastication, swallowing, aesthetics, and pronunciation are seriously affected. At present, the common repair methods are mainly non-physiological repair methods, including fixed dentures, removable dentures, and implant dentures, which can restore the shape and some functions of the teeth. But they still have various problems. So the ideal repair method is physiological tooth regeneration, including periodontal ligament regeneration, pulp–dentin complex regeneration, bioengineered tooth root (bio-root) regeneration, and whole-tooth regeneration (Oshima and Tsuji, 2015; Gao et al., 2016; Wu et al., 2019). For bio-root regeneration, the key factors which affect its effect include seed cells, scaffold materials, growth factors, etc. (Ramanathan et al., 2018). Studies verified that dental pulp stem cells (DPSCs) are good seed cells for regeneration of bio-root. Compared with periodontal ligament stem cells (PDLSCs) and bone marrow stem cells (BMSCs), DPSCs have a stronger proliferation ability, mineralization ability, and anti-aging ability (Yu et al., 2007; Huang et al., 2009; Iezzi et al., 2019; Ma et al., 2019). Although some research achievements have been made, the known regulatory mechanisms cannot effectively induce the odontogenic differentiation of mesenchymal stem cells (MSCs). It inhibited the application for bio-root regeneration. Therefore, it is very important to further clarify the regulation mechanism of odontogenic differentiation in DPSCs.

Epigenetics refers to the heritability change of differential gene expression under the condition of the same DNA sequence, including DNA methylation, histone modification, RNA methylation, and non-coding RNA (ncRNA) (Mortada and Mortada, 2018; Sui et al., 2020). Among them, the modification of non-coding RNA performs an significant role in the odontogenic differentiation of MSCs (Ju et al., 2019; Wang et al., 2019; Zheng et al., 2019). miRNA is a small non-coding RNA with a length of about 22 nucleotides. The function of miRNA was to silence the downstream target gene by binding to its 3′UTR region (Strubberg and Madison, 2017). Previous investigations show that miRNA plays an important role in the odontogenic differentiation of MSCs (Bao et al., 2020; Liu et al., 2020). Our studies have found that there is a significant difference of some miRNA between dental tissue-derived MSCs including DPSCs and non-dental tissue-derived MSCs. And the expression of miR-6807-5p in dental tissue-derived MSCs was significantly higher than that in non-dental tissue-derived MSCs. An existing study indicates that miR-6807-5p mainly involved in the inflammatory process by downregulating drug metabolism cytochrome P450 (Kugler et al., 2020). In addition, miR-6807-5p was also one of the biomarkers of gastric cancer (Iwasaki et al., 2019). However, the role of miR-6807-5p in odontogenic differentiation of DPSCs has not been elucidated. In this study, we will explore the function and mechanism of miR-6807-5p in the odontogenic differentiation of DPSCs.

RNA methylation is a reverse modification at the RNA level, which is extremely important in epigenetic modification (Li et al., 2020). And m6A modification is the most abundant RNA internal modification in eukaryotic cells (Chen et al., 2021). m6A methylation is mediated by a multi-protein complex including METTL3, METTL14, and WTAP, and demethylase is mainly mediated by two m6A demethylated transferases FTO and ALKBH5 (Cao et al., 2016). A previous study shows that m6A modification plays an important role in the formation of the root of the tooth (Sheng et al., 2021). Methyltransferase like 7A (METTL7A) belongs to the human methyltransferase-like protein (METTL) family, which is characterized by the presence of the *S*-adenosylmethionine domain (Ignatova et al., 2019). It has been found that METTL7A promoted the viability and osteogenic differentiation of BMSCs by changing the methylation state of related genes (Lee et al., 2021). An existing study indicates that METTL7A is mainly involved in the formation of lipid droplets and mental diseases (Zhou et al., 2017). The differential expression of METTL7A leads to frontotemporal dementia (Taskesen et al., 2017). In addition, the abnormal expression of METTL7A may affect thyroid cancer, choriocarcinoma, and breast cancer (Zhou et al., 2017; Jun et al., 2020; Yang et al., 2021). By software prediction, METTL7A may be the downstream target gene of miR-6807-5p. Whether METTL7A is involved in miR-6807-5p regulation and influenced the odontogenic differentiation of DPSCs is unclear and needs to be further studied.

In this study, we intend to explore the function and mechanism of miR-6807-5p and METTL7A in the odontogenic differentiation of DPSCs. Our study found that miR-6807-5p inhibited the odontogenic differentiation of DPSCs through directly targeting METTL7A.

## Materials and Methods

### Cell Cultures

The DPSCs were obtained from the third molars extracted from patients in Beijing Stomatological Hospital Affiliated to Capital Medical University. Informed consent of patients was obtained. The extracted third molars were immersed in sterile PBS and isolated under sterile conditions as in previous studies (Shi et al., 2019). Pulp tissue was removed from the broken tooth. The pulp tissue was cut up with scissors and then rinsed with penicillin–streptomycin. After digestion with collagenase type I (Invitrogen, Carlsbad, CA, United States) and dispase (Invitrogen) for 1 h at 37°C, the pulp tissue was inoculated into a dish. The fluid was changed 7 days later. Passages 3–5 of DPSCs were used in this study. Human embryonic kidney 293T cells (HEK293T, American Type Culture Collection, Manassas, VA, United States) were cultured in DMEM complete medium containing 10% FBS (Invitrogen), 100 U/ml penicillin and 100 μg/ml streptomycin (Invitrogen).

### Construction of Plasmid and Transfection of Virus

The lentiviruses of Consh, METTL7Ash, miR-6807-5p inhibitor/mimic, and SNRNP200sh were purchased from the GenePharma Company (Suzhou, China). An HA tag was combined with the full-length sequence of human METTL7A and constructed on the retroviral vector pQCXIN (Taihe Biotechnology Co., Ltd., Beijing, China). The HA-METTL7A plasmid and package vectors were transfected into 293T cells. After 24 h, 100 μl sodium butyrate was added. After 72 h, the retrovirus was collected. As in the previous study (Yang et al., 2020a), DPSCs were plated overnight. The DPSCs were transfected with the lentivirus for 12 h. And the transfected DPSCs were purified by puromycin for 3 days. The DPSCs were transfected with retrovirus for 12 h. And the transfected DPSCs were purified by G418 for 10 days.

### RT-PCR and Real-Time RT-PCR

The TRIzol reagent (Invitrogen) was used to isolate all RNAs from DPSCs. The RT-PCR experiment was performed according to the instructions of the reverse-transcription kit (Vazyme #R333, Nanjing, China). And real-time RT-PCR was used to evaluate the gene expression based on the methods described in a previous study (Yang et al., 2020c). Supplementary Table 1 shows the primers for specific genes.

### Western Blot

As in the previous method (Yang et al., 2020b), the RIPA buffer was used to lyse the cells. After quantitative denaturation, SDS-PAGE and appropriate antibody were used to detect the expression of related proteins. The primary antibody is a monoclonal antibody: anti-METTL7A (Cat No. ab79207, Abcam, Cambridge, United Kingdom), anti-SNRNP200 (Cat No. ab241589, Abcam), anti-BSP (Cat No. ab52128, Abcam), anti-OCN (Cat No. ab133612, Abcam), anti-OSX (Cat No. ab209484, Abcam). The housekeeping protein was detected with monoclonal glyceraldehyde 3-phosphate dehydrogenase antibody (GAPDH Cat No. C1312, Applygen Technologies, Beijing, China) and histone H3 antibody (Cat No. SC-56616, Santa Cruz Biotechnology, Santa Cruz, CA, United States).

### Alkaline Phosphatase Activity Assay and Alizarin Red Staining Detection

DPSCs were cultured in a mineralized-inducing medium. After 5 days of induction, ALP activity was detected by an ALP activity kit (Sigma-Aldrich, St. Louis, MO, United States). After 2 weeks of induction, the mineralization was detected by ARS and calcium ion quantification. These experiments followed the same protocols as the previous ones (Yan et al., 2019).

### Co-immunoprecipitation

As in the previous experimental method (Yang et al., 2020a), we used the IP lysis buffer (Invitrogen) to lyse the cells. Cell lysates were incubated with a specific antibody for 4 h and then added with protein A/G Sepharose (Santa Cruz, Biotechnology, United States) or HA tag beads (Thermo Scientific Pierce, United States) and rotated overnight at 4°C. With the help of magnetic support, the beads were washed three times with PBS and were resuspended in an SDS-PAGE loading buffer for western blotting analysis using a corresponding antibody. The primary antibodies were as follows: anti-SNRNP200 (Cat No. ab241589, Abcam), anti-METTL7A (Cat No. ab79207, Abcam), anti-IgG (Cat No. bs-0297P, Bioss, Beijing, China).

### Pull-Down Assay With Biotinylated miRNA

As in a previous experimental protocol (Wang et al., 2015), the DPSCs were transfected with biotinylated miR-6807-5p (50 nM). The transfected DPSCs were harvested after 48 h. The lysates were incubated with M-280 streptavidin magnetic beads (Sigma-Aldrich) at 4°C for 2 h. After that, part of them was added with TRIzol to extract RNA. And then the gene expression was detected by real-time RT-PCR. Another part of them was added with a loading buffer to extract protein. The possible co-binding protein was detected by protein mass spectrometry.

### Dual-Luciferase Reporter Assay

Wild-type or mutant-type METTL7A 3′UTR-Luc reporter constructs and miR-6807-5p mimic or miR-NC were co-transfected into 293T cells. After 48 h, the 293T cells were harvested. Then, the dual-luciferase reporter assay kit was used to detect the activity (E1910, Promega, Madison, WI, United States).

### Mass Spectrometry Analysis

The protein was separated by SDS-PAGE. And then it was stained with a Fast Silver Stain Kit (Beyotime Biotechnology, Shanghai, China). Finally, the gel band was analyzed by mass spectrometry.

### Transplantation in Nude Mice

The animal study was authorized by the Animal Care Institution and carried out according to the animal experiment ordinance of the Beijing Stomatological Hospital Affiliated to Capital Medical University. Nude mice (8-week-old female, nu/nu) were purchased from the Institute of Animal Science Research of the Vital River Co., Ltd. AS in the previous study (Yang et al., 2020c), 2 × 10^6^ cells of DPSCs-miR-6807-5p inhibitor and DPSCs-Consh were mixed with 20 mg HA/tricalcium phosphate (Engineering Research Center for biomaterials, Chengdu, China). Then, all of them were transplanted subcutaneously into the back of nude mice. After 8 weeks, samples were collected and then stained with HE. Image Pro Plus analysis was used to calculate the area of mineralized tissue.

### Immunohistochemistry Staining

As in the previous study (Yang et al., 2020c), 5-μm tissue sections were decarbonized and rehydrated. The tissue sections were treated with the antigen retrieval method, followed by quenching the endogenous peroxidase activity for 10 min. The sections were incubated with the first antibody at 4°C overnight and finally stained. The main antibodies were as follows: anti-DSPP (Cat No. bs-10316R, Bioss), anti-DMP1 (Cat No. bs-12359R, Bioss), anti-BSP (Cat. No. ab52128, Abcam).

### Statistical Analyses

SPSS version 22 statistical software was used for statistical calculation. Statistical significance was determined by Student’s *t*-test or Tukey’s *post hoc* tests used after one-way ANOVA, with *p* ≤ 0.05 considered significant.

## Results

### miR-6807-5p Inhibited the Odontogenic Differentiation of Dental Pulp Stem Cells

**FIGURE 1:**
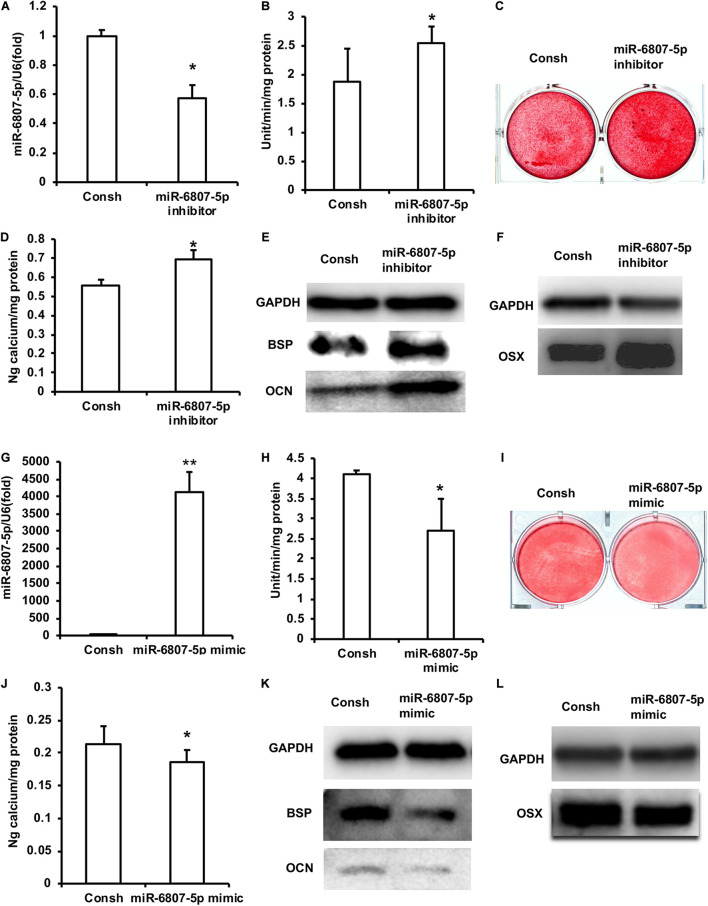
miR-6807-5p inhibited the odontogenic differentiation of DPSCs. **(A)** The expression of miR-6807-5p was detected by real-time RT-PCR. **(B)** ALP activity after 5 days of mineralized induction. **(C,D)** ARS and Calcium ion quantification results after 2 weeks of mineralized induction. **(E)** After 1 week of mineralized induction, the expressions of BSP and OCN were detected by western blot. **(F)** The expression of OSX was detected by western blot. **(G)** The expression of miR-6807-5p was detected by real-time RT-PCR. **(H)** ALP activity after 5 days of induction. **(I,J)** ARS and calcium ion quantitative results after 2 weeks of induction. **(K)** After 1 week of induction, the expressions of BSP and OCN were detected by western blot. **(L)** The expression of OSX was detected by western blot. GAPDH was used as internal control. Student’s *t*-test was performed to determine statistical significance. All error bars represent the standard deviation (*n* = 3). *p ≤ 0.05, **p ≤ 0.01.

In a previous study, the DPSCs have been validated by flow cytometry analysis (Yi et al., 2017). In order to clarify the function of miR-6807-5p in DPSCs, we transfected DPSCs with the lentivirus of Consh and miR-6807-5p inhibitor. After the infected DPSCs were treated with puromycin for 3 days, the expression of miR-6807-5p in the miR-6807-5p inhibitor group was lower than that in the control group detected by real-time RT-PCR (Figure 1A). Then, the DPSCs were cultured in mineralized-inducing mediums. After 5 days of induction, it was found that the ALP activity in the miR-6807-5p inhibitor group was higher than that in the control group (Figure 1B). After 2 weeks of induction, the results of ARS and calcium ion quantification showed that the mineralization was significantly increased in the miR-6807-5p inhibitor compared with the control group (Figures 1C,D). After 1 week of induction, the expressions of BSP and OCN in the miR-6807-5p inhibitor group were significantly higher than those in the control group detected by western blot (Figure 1E). The western blot results showed that OSX was also significantly higher in the miR-6807-5p inhibitor group than in the control group (Figure 1F).

Then we transfected DPSCs with the lentivirus of Consh and miR-6807-5p mimic. After 3 days of puromycin screening, the expression of miR-6807-5p was significantly increased in the miR-6807-5p mimic group compared with the control group detected by real-time RT-PCR (Figure 1G). Then, the transfected DPSCs were cultured in mineralized-inducing mediums. After 5 days of induction, the ALP activity in the miR-6807-5p mimic group was significantly lower than that in the control group (Figure 1H). After 2 weeks of induction, ARS and calcium ion quantification results showed that the mineralization in the miR-6807-5p mimic group was significantly lower than that in the control group (Figures 1I,J). After 1 week of induction, the western blot results showed that the expressions of BSP and OCN in the miR-6807-5p mimic group were significantly lower than those in the control group (Figure 1K). The expression of OSX was also significantly lower in the miR-6807-5p mimic group than in the control group as detected by western blot (Figure 1L).

**FIGURE 2:**
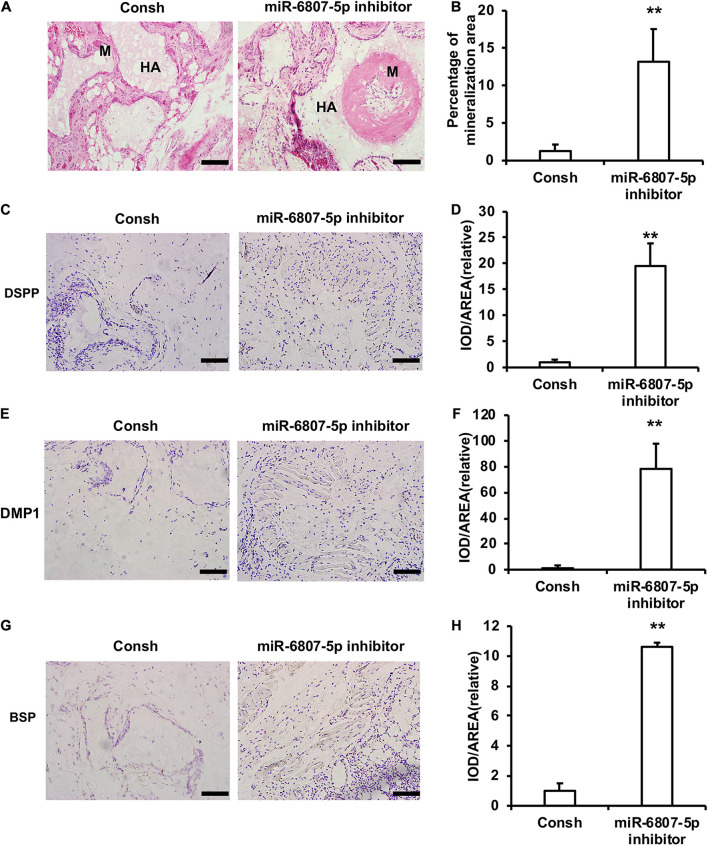
miR-6807-5p inhibited the mineralization tissue formation mediated by DPSCs *in vivo*. **(A)** The result of HE staining showed the mineralized tissue. Scale bar: 100 μm. M: Mineralized tissue, HA: Hydroxyapatite tricalcium carrier. **(B)** The quantitative analysis of HE staining. **(C–H)** Immunohistochemical staining and quantitative analysis of DSPP **(C,D)**, DMP1 **(E,F)**, and BSP **(G,H)**. Student’s *t*-test was performed to determine statistical significance. All error bars represent the standard deviation (*n* = 5). **p ≤ 0.01.

Moreover, the DPSCs transfected with the lentivirus of Consh and miR-6807-5p inhibitor were mixed with HA/TCP material and then transplanted into nude mice for 8 weeks. The results of HE staining and quantitative measurements of the mineralization tissue area showed that the amount of mineralization tissue in the miR-6807-5p inhibitor group was significantly increased compared with that in the control group (Figures 2A,B). The immunohistochemical staining results showed that the expressions of DSPP, DMP1, and BSP in the miR-6807-5p inhibitor group were significantly increased compared with the those in the control group (Figures 2C,E,G). The quantitative measurements of immunohistochemical staining results showed that the expressions of the DSPP, DMP1, and BSP in the miR-6807-5p inhibitor group were increased compared with those in the control group (Figures 2D,F,H).

### METTL7A Was Identified as the Downstream Target Gene of miR-6807-5p

In order to further clarify the mechanism of miR-6807-5p in the odontogenic differentiation, we performed the pull-down assay with biotinylated miRNA and mass spectrometry analysis. The results showed that there were 10 possible co-binding proteins of miR-6807-5p including MYH9, PLEC, and MYO1C (Supplementary Table 2).

**FIGURE 3:**
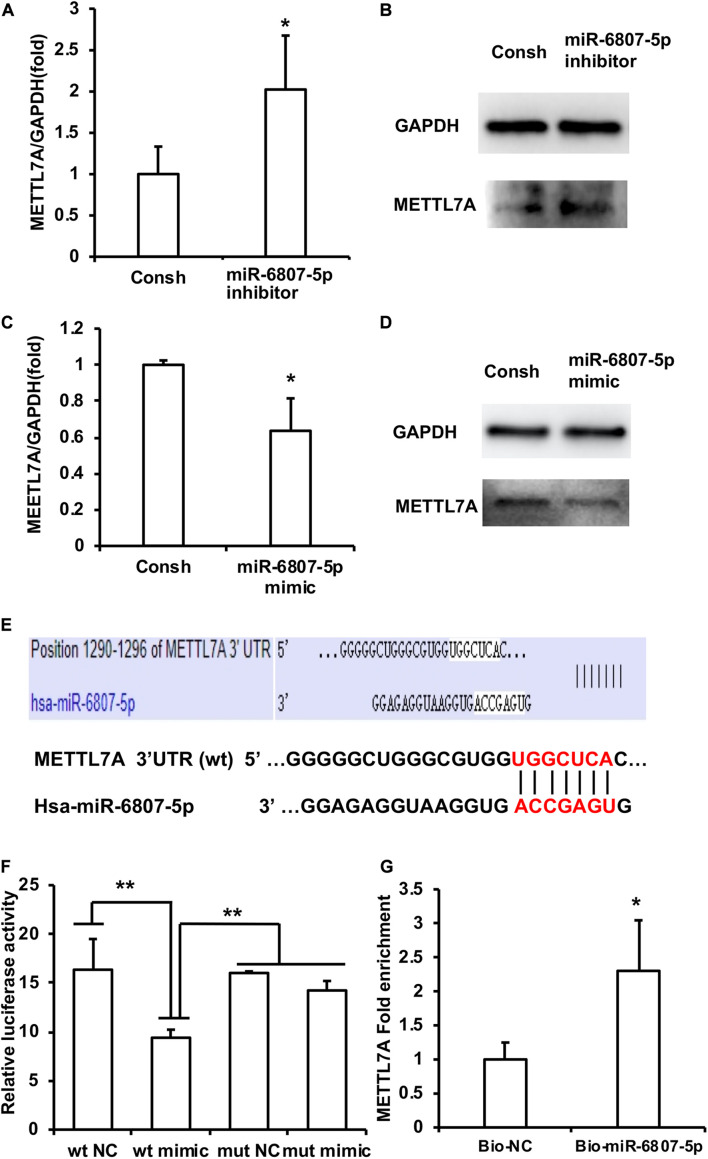
METTL7A was identified as the downstream target gene of miR-6807-5p. **(A,B)** METTL7A was promoted in the miR-6807-5p inhibitor group detected by real-time RT-PCR and western blot. **(C,D)** METTL7A was repressed in the miR-6807-5p mimic group detected by real-time RT-PCR and western blot. **(E)** The binding site of miR-6807-5p in 3′UTR of METTL7A. **(F)** The miR-6807-5p binding to the 3′UTR of METTL7A was verified by the dual-luciferase reporter assay. **(G)** Results of the pull-down assay with biotinylated miRNA. GAPDH was used as internal control. Student’s *t*-test or Tukey’s *post hoc* tests used after one-way ANOVA were performed to determine statistical significance. All error bars represent the standard deviation (*n* = 3). *p ≤ 0.05, **p ≤ 0.01.

Then, the downstream target genes of miR-6807-5p were predicted by miRBase software. It was predicted that GREM1, TRMT10C, METTL1, and METTL7A may be the downstream target genes of miR-6807-5p. Then, these candidate genes were investigated by real-time RT-PCR. The real-time RT-PCR results showed that only METTL7A expression was increased in the miR-6807-5p inhibitor group compared with the control group (Figure 3A and Supplementary Figure 1). Western blot results confirmed that the expression of METTL7A was increased in the miR-6807-5p inhibitor group (Figure 3B). And further results showed that METTL7A was decreased in the miR-6807-5p mimic group compared with the control group detected by real-time RT-PCR and western blot (Figures 3C,D). Further, the binding sites of miR-6807-5p in the 3′UTR of METTL7A were predicted by miRBase software (Figure 3E). Then, the double-luciferase reporter assay was used and verified the binding site of miR-6807-5p in the 3′UTR of METTL7A (Figure 3F). Moreover, after pull-down with biotinylated miRNA, the real-time RT-PCR results showed that the amount of METTL7A in the Bio-miR-6807-5p group was enhanced compared with that in the control group (Figure 3G).

### METTL7A Promoted the Odontogenic Differentiation of Dental Pulp Stem Cells

**FIGURE 4:**
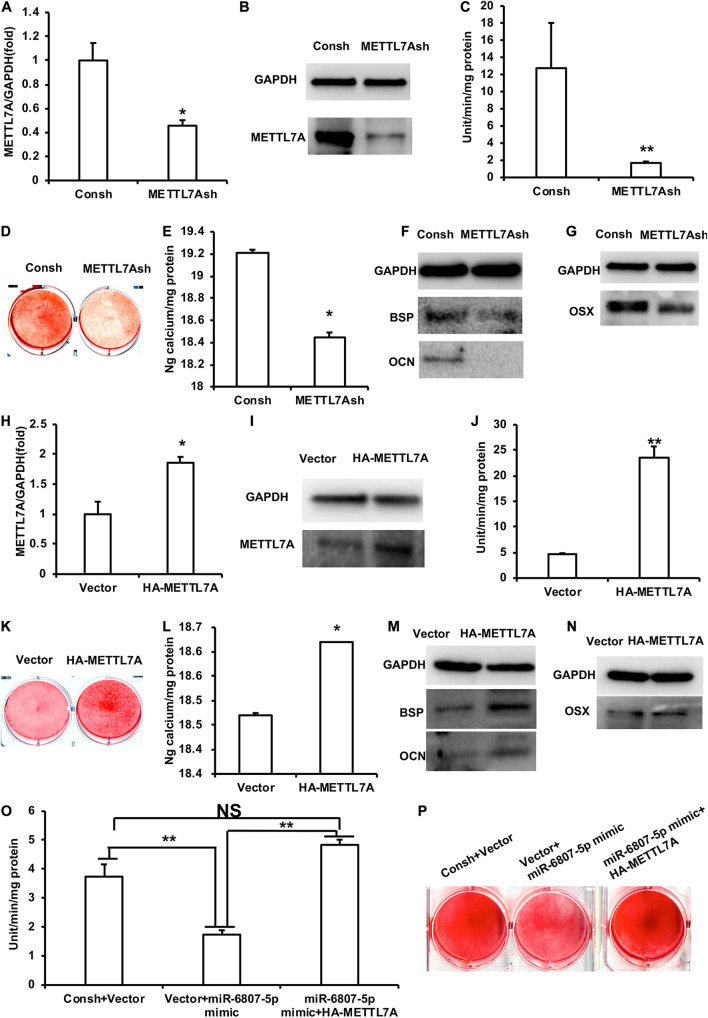
METTL7A promoted the odontogenic differentiation of DPSCs. **(A,B)** The expression of METTL7A was detected by real-time RT-PCR and western blot. **(C)** ALP activity after 5 days of mineralized induction. **(D,E)** ARS and calcium ion quantification after 2 weeks of induction. **(F)** The expressions of BSP and OCN were detected by western blot after 1 week of induction. **(G)** The expression of OSX was weakened in the METTL7Ash group compared with that in the control group detected by western blot. **(H,I)** The expression of METTL7A was detected by real-time RT-PCR and western blot. **(J)** ALP activity after 5 days of mineralized induction. **(K,L)** ARS and calcium ion quantification after 2 weeks of mineralized induction. **(M)** The expressions of BSP and OCN were detected by western blot after 1 week of induction. **(N)** The expression of OSX was detected by western blot. **(O)** ALP activity of the Consh + Vector, Vector + miR-6807-5p mimic, and miR-6807-5p mimic + HA-METTL7A groups after 5 days of mineralized induction. **(P)** ARS of the Consh + Vector, Vector + miR-6807-5p mimic, and miR-6807-5p mimic + HA-METTL7A groups after 2 weeks of mineralized induction. GAPDH was used as internal control. Student’s *t*-test or Tukey’s *post hoc* tests used after one-way ANOVA was performed to determine statistical significance. All error bars represent the standard deviation (*n* = 3). *p ≤ 0.05, **p ≤ 0.01.

In order to detect the function of METTL7A in the process of odontogenic differentiation of DPSCs, we transfected the DPSCs with the lentivirus of Consh and METTL7Ash. After the transfected DPSCs were purified by puromycin for 3 days, real-time RT-PCR and western blot results showed that the expression of METTL7A in DPSCs-METTL7Ash was lower than that in DPSCs-Consh (Figures 4A,B). Then, the transfected DPSCs were cultured in mineralized-inducing mediums. After 5 days of induction, the ALP activity in the DPSCs-METTL7Ash group was lower than that in DPSCs-Consh group (Figure 4C). After 2 weeks of induction, ARS and calcium ion quantification results showed that the mineralization in the DPSCs-METTL7Ash group was lower than that in the DPSCs-Consh group (Figures 4D,E). After 1 week of induction, western blot results showed that the expressions of BSP and OCN in the DPSCs-METTL7Ash group were significantly lower than those in DPSCs-Consh group (Figure 4F). Western blot results also showed that the expression of OSX in the DPSCs-METTL7Ash group was lower than that in the DPSCs-Consh group (Figure 4G).

Then, we transfected DPSCs with the retrovirus of vector and HA-METTL7A. After the transfected DPSCs were purified by G418 for 10 days, the expression of METTL7A was higher in the DPSCs-HA-METTL7A than in the DPSCs-Vector detected by real-time RT-PCR and western blot (Figures 4H,I). Then, the transfected DPSCs were cultured in mineralized-inducing mediums. After 5 days of induction, the results of ALP activity showed that the mineralization in the DPSCs-HA-METTL7A was significantly higher than that in the DPSCs-Vector (Figure 4J). After 2 weeks of induction, ARS and calcium ion quantification results revealed that the overexpression of METTL7A promoted the mineralization of DPSCs compared with the control group (Figures 4K,L). After 1 week of induction, western blot results showed that the expressions of BSP and OCN in the DPSCs-HA-METTL7A group were significantly higher than those in the DPSCs-Vector group (Figure 4M). Western blot results also showed that the expression of OSX in the HA-METTL7A group was significantly higher than that in the control group (Figure 4N).

In order to further verify that miR-6807-5p affected the odontogenic differentiation of DPSCs by silencing METTL7A, we designed a rescue experiment. The DPSCs were transfected with the lentivirus and retrovirus of Consh + Vector, Vector + miR-6807-5p mimic, and miR-6807-5p mimic + HA-METTL7A. Then, the transfected DPSCs were cultured in mineralized-inducing mediums. After 5 days of induction, the ALP activity in the DPSCs-Vector + miR-6807-5p mimic group was lower than that in the DPSCs-Consh + Vector and DPSCs-miR-6807-5p mimic + HA-METTL7A groups. There is no significant difference between the DPSCs-Consh + Vector group and the DPSCs-miR-6807-5p mimic + HA-METTL7A group (Figure 4O). After 2 weeks of induction, ARS results revealed that the mineralization in the DPSCs-Vector + miR-6807-5p mimic group was lower than that in the DPSCs-Consh + Vector and DPSCs-miR-6807-5p mimic + HA-METTL7A groups. And there is no significant difference between the DPSCs-Consh + Vector group and the DPSCs-miR-6807-5p mimic + HA-METTL7A group (Figure 4P).

### SNRNP200 Was Identified as a Co-binding Protein of METTL7A and Promoted the Odontogenic Differentiation of Dental Pulp Stem Cells

**FIGURE 5:**
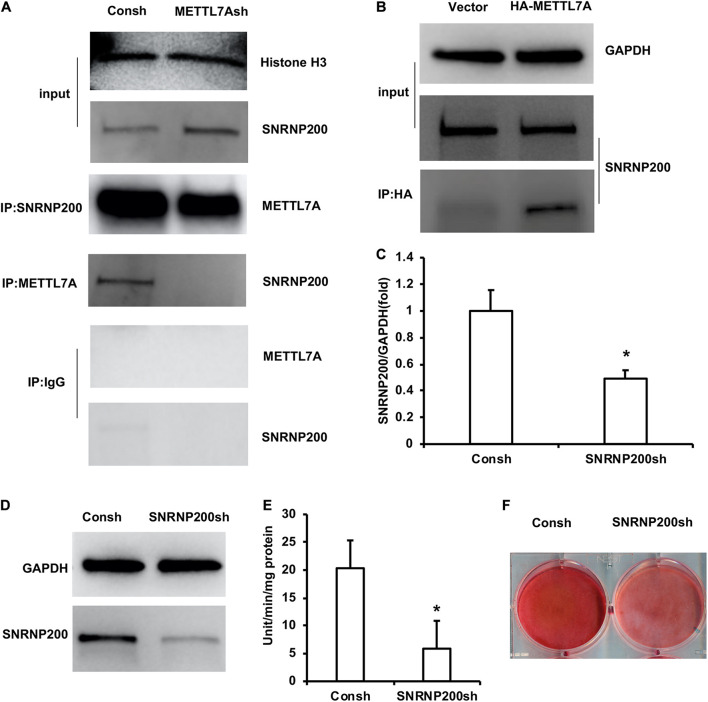
SNRNP200, identified as a co-binding protein of METTL7A, promoted odontogenic differentiation of DPSCs. **(A)** The association of SNRNP200 and METTL7A in METTL7A-knockdown DPSCs detected by the Co-IP assay. **(B)** The association of SNRNP200 and METTL7A in METTL7A-overexpressed DPSCs detected by the Co-IP assay. **(C,D)** The expression of SNRNP200 was detected by real-time RT-PCR and western blot. **(E)** ALP activity after 5 days of mineralized induction. **(F)** ARS after 2 weeks of induction. GAPDH and histone H3 were used as internal control. Student’s *t*-test was performed to determine statistical significance. All error bars represent the standard deviation (*n* = 3). *p ≤ 0.05.

In order to further study the mechanism of METTL7A in the odontogenic differentiation of DPSCs, protein mass spectrometry was performed to identify the binding partner of METTL7A. The protein mass spectrometry results showed that there are 11 possible co-binding proteins of METTL7A including SNRNP200, AGO1, WDR1, and so on (Supplementary Table 3). Then we selected the candidate co-binding protein of METTL7A, SNRNP200, by analyzing the molecule weight of protein and the amount of peptide. Further, the Co-IP results showed that the association of SNRNP200 and METTL7A was decreased in the DPSCs-METTL7Ash group compared with that in the DPSCs-Consh group (Figure 5A). And the Co-IP assay also showed that the association of SNRNP200 and METTL7A was increased in the DPSCs-HA-METTL7A group compared with that in the DPSCs-Vector group (Figure 5B).

The DPSCs were transfected with the lentivirus of Consh and SNRNP200sh. After the transfected DPSCs were purified by puromycin for 3 days, real-time RT-PCR and western blot results showed that the expression of SNRNP200 was weakened in the DPSCs-SNRNP200sh group compared with that in the control group (Figures 5C,D). Then, the transfected DPSCs were cultured in mineralized-inducing mediums. After 5 days of induction, the ALP activity in the DPSCs-SNRNP200sh was significantly repressed compared with that in the control group (Figure 5E). After 2 weeks of induction, the ARS results revealed that the mineralization in the DPSCs-SNRNP200sh group was significantly repressed compared with that in the control group (Figure 5F).

## Discussion

With the development of tissue engineering, tooth tissue regeneration mediated by MSCs has become the most promising and ideal repair method. Therefore, it is very important to clarify the regulation method for odontogenic differentiation in seed cells.

In this study, ALP, ARS, and Calcium ion quantification showed that miR-6807-5p inhibited the mineralization of DPSCs. Western blot results showed that BSP and OCN, key indicators of osteogenesis/odontogenesis, were inhibited by miR-6807-5p. BSP localizes to the matrix of mineralization and involved in the formation of hydroxyapatite crystal in the bone and tooth (Zhang et al., 2009). OCN appears as an indicator at the end stage of osteoblast differentiation. It can regulate calcium homeostasis and osteogenic mineralization (An et al., 2016). The process of odontogenesis is highly similar to osteogenesis (Bosshardt, 2005). And the process of cementum formation by cementoblasts is similar to that of bone formation. In addition, to form mineralized nodules *in vitro*, cementoblasts express genes such as BSP and OCN (D’Errico et al., 2000; Wang et al., 2016). These results confirmed that miR-6807-5p inhibited the odontogenic differentiation of DPSCs. In order to investigate the expression of the key transcription factor of odontogenic differentiation, we detected the expression of OSX in DPSCs. OSX, also known as Sp7, is a key transcription factor in the osteogenic/odontogenic differentiation of MSCs. Studies revealed that OSX-inactivated mice had no bone formation (Nakashima et al., 2002; Baek and Kim, 2011). In this study, we found that miR-6807-5p inhibited the expression of OSX. In order to further verify, the function of miR-6807-5p, we performed the experiment of transplantation in nude mice. The *in vivo* result showed that miR-6807-5p inhibited the mineralization tissue formation and the expressions of DSPP and DMP1. DSPP and DMP1, two major phosphoproteins of non-collagenous proteins, can regulate dentin regulation (Liu Z. et al., 2021). DSPP and DMP1 are highly seen in odontoblast and dentine during tooth development and formation (Liu M.M. et al., 2021). Therefore, these results confirmed that miR-6807-5p inhibited the odontogenic differentiation of DPSCs.

In order to further study the mechanism of miR-6807-5p in the odontogenic differentiation of DPSCs, we performed protein mass spectrometry to find the co-binding protein of miR-6807-5p. The results identified that there were 10 possible co-binding proteins of miR-6807-5p including MYH9 and MYO1C. MYH9 and MYO1C are associated with bone formation and absorption (Desh et al., 2014; Kanzaki et al., 2017). Existing studies have not reported these proteins related to tooth formation. Thus, the role of the candidate co-binding protein of miR-6807-5p in odontogenic differentiation needs to be further studied.

Then we predicted the target genes of miR-6807-5p to further explore the mechanism of miR-6807-5p in odontogenic differentiation of DPSCs and identified four possible downstream targets including GREM1, TRMT10C, METTL1, and METTL7A. GREM1, an antagonist of bone morphogenetic protein-2 (BMP-2), can specifically bind to BMP-2 to block its function. It is necessary for bone development and maintenance of bone homeostasis (Canalis et al., 2012). TRMT10C, which encodes the precursor of the mitochondrial ribonuclease P subunit, is involved in the transcription of tRNA, RNA processing, and mitochondrial respiration (Karasik et al., 2021). METTL1 plays an important role in the regulation of mRNA output, splicing, and translation by affecting the modification of m7G RNA (Deng et al., 2020). METTL7A, methyltransferase-like 7A, is an integrated membrane protein anchored to the endoplasmic reticulum. Recent studies have found that METTL7A affected the survival ability and osteogenic differentiation of BMSCs by affecting the methylation status of some related genes (Lee et al., 2021). METTL7A is also a tumor suppressor gene with decreased expression in osteosarcoma (Jia et al., 2021). Moreover, these gene expressions were detected by real-time RT-PCR and western blot. It was found that only METTL7A was upregulated in the miR-6807-5p inhibitor group and downregulated in the miR-6807-5p mimic group. We also confirmed that miR-6807-5p bound to the 3′UTR region of the downstream target gene METTL7A by the dual-luciferase reporter assay and pull-down assay with biotinylated miRNA. Furthermore, the results of ALP, ARS, and calcium ion quantification and the expressions of BSP, OCN, and OSX showed that METTL7A promoted the odontogenic differentiation of DPSCs. And METTL7A could rescue the ALP activity and mineralization ability in DPSCs, which are impaired by miR-6807-5p, indicating that miR-6807-5p inhibited the odontogenic differentiation of DPSCs via negatively regulating METTL7A.

In order to study the mechanism of METTL7A in the odontogenic differentiation of DPSCs, we analyzed the co-factors of METTL7A by protein mass spectrometry. We found 11 possible co-binding proteins of METTL7A including SNRNP200, AGO1, and WDR1. By comparing the molecular weight of protein and the amount of peptide, we found that SNRNP200 may be the candidate co-factor of METTL7A in DPSCs. SNRNP200 belongs to the pre-nuclear splicing gene. It is essential for the splicing of pre-miRNA. The defect of SNRNP200 may interfere in the recognition of transcripts, resulting in an abnormal splicing product (Absmeier et al., 2016). It is found that the mutation of SNRNP200 leads to retinopathy retinitis pigmentosa (Benaglio et al., 2011; Liu et al., 2012; Zhang et al., 2013; Cvačková et al., 2014). A study also revealed that SNRNP200 is also involved in the antiviral response (Tremblay et al., 2016). In acute myeloid leukemia, U-SNRNP200 complex was found to be exposed to the leukemia cell membrane, which provides a target for tumor therapy (Gillissen et al., 2018). In addition, SNRNP200 is also involved in the process of mammalian cell cycle (Ehsani et al., 2013). In terms of osteogenesis, it has been previously reported that SNRNP200 knockdown can inhibit the osteogenic/odontogenic differentiation of SCAPs (Su et al., 2020). In this study, we found that knockdown of SNRNP200 inhibited the ALP activity and mineralization ability in DPSCs, indicating that SNRNP200 might promote the odontogenic differentiation of DPSCs. Therefore, we speculated that the SNRNP200 and METTL7A protein complex may affect the splicing of the key gene involving the odontogenic differentiation by influencing the methylation process of m6A. And thus these two proteins synergistically promoted the odontogenic differentiation of DPSCs. This speculation and regulation mechanism need to be further studied.

## Conclusion

In conclusion, this study verified that miR-6807-5p inhibited the odontogenic differentiation of DPSCs via directly targeting METTL7A and repressing its expression. METTL7A might promote the odontogenic differentiation of DPSCs by associating with SNRNP200. This investigation provided the candidate targets for enhancing the odontogenic differentiation of dental MSCs and dental tissue regeneration.

## Data Availability Statement

The original contributions presented in the study are included in the article/Supplementary Material, further inquiries can be directed to the corresponding author/s.

## Ethics Statement

The studies involving human participants were reviewed and approved by the Ethical Committee of Beijing Stomatological Hospital (Review No. 2011–02). The patients/participants provided their written informed consent to participate in this study. The animal study was reviewed and approved by Animal experiments were approved by the Beijing Stomatological Hospital, Capital Medical University (Ethics Committee Agreement, Beijing Stomatological Hospital Ethics Review No. KQYY-201804-003).

## Author Contributions

NW: responsible for data collection and collation, interpretation, and manuscript writing. XH and HY: responsible for data collection and collation. ZF and DX: responsible for conception, design, manuscript revision, confirmation, and so on. All authors contributed to the article and approved the submitted version.

## Conflict of Interest

The authors declare that the research was conducted in the absence of any commercial or financial relationships that could be construed as a potential conflict of interest.

## Publisher’s Note

All claims expressed in this article are solely those of the authors and do not necessarily represent those of their affiliated organizations, or those of the publisher, the editors and the reviewers. Any product that may be evaluated in this article, or claim that may be made by its manufacturer, is not guaranteed or endorsed by the publisher.

## Supplementary Material

Supplementary Figure 1 The gene expressions were detected by real-time RT-PCR. **(A–C)** There was no significant difference of the expression of GREM1 **(A)**, TRMT10C **(B)**, and METTL1 **(C)** in the miR-6807-5p inhibitor group compared with the control group. GAPDH was used as internal control. Student’s *t*-test was performed to determine statistical significance. All error bars represent the standard deviation (*n* = 3).
