# Supplementary material for: miR-6807-5p Inhibited the Odontogenic Differentiation of Human Dental Pulp Stem Cells Through Directly Targeting METTL7A
Source: Front Cell Dev Biol. 2021 Nov 1;9:759192. doi: 10.3389/fcell.2021.759192 (PMC8591228; doi:10.3389/fcell.2021.759192)
Supplement: Supplementary file 1 [file Table_1.DOCX]

**Supplementary Table 1. Primer Sequences used for Real-time RT-PCR**

| **Gene** | **Primer sequence （5’-3’）** |
| --- | --- |
| GAPDH | F- cggaccaatacgaccaaatccg  R- agccacatcgctcagacacc |
| METTL7A | F- tgcagggtgacctgtattga  R- actgcaggtgtcggttctct |
| SNRNP200 | F-ctgccacacttcacctctga  R-tccatctccatgatgtcgaa |
| GREM1 | F-gtcacactcaactgccctga  R-cgatggatatgcaacgacac |
| TRMT10C | F- tggtgccatttacccttcat  R- agctcttcagaagggggtgt |
| METTL1 | F-gggacatctaggcacctcaa  R-ctggaggacgggatcttgta |
