# Supplementary material for: miR-6807-5p Inhibited the Odontogenic Differentiation of Human Dental Pulp Stem Cells Through Directly Targeting METTL7A
Source: Front Cell Dev Biol. 2021 Nov 1;9:759192. doi: 10.3389/fcell.2021.759192 (PMC8591228; doi:10.3389/fcell.2021.759192)
Supplement: Supplementary file 2 [file Table_2.DOCX]

**Supplementary Table 2. Protein-mass spectrometry results of miRNA pull down**

| Accession | Name | Species | Peptides(95%) |
| --- | --- | --- | --- |
| NP_002464 | myosin heavy chain 9 | HUMAN | 4 |
| NP_000436 | plectin | HUMAN | 3 |
| NP_001374773 | proline rich coiled-coil 2C | HUMAN | 2 |
| NP_001185844 | PPFIA binding protein 1 | HUMAN | 1 |
| NP_060101 | DExD/H-box helicase 60 | HUMAN | 1 |
| NP_001123630 | myosin IB | HUMAN | 1 |
| NP_001074248 | myosin IC | HUMAN | 1 |
| NP_001193496 | interferon gamma inducible protein 16 | HUMAN | 1 |
| NP_000108 | Emerin | HUMAN | 1 |
| NP_001128711 | lactate dehydrogenase A | HUMAN | 1 |
