# Supplementary material for: miR-6807-5p Inhibited the Odontogenic Differentiation of Human Dental Pulp Stem Cells Through Directly Targeting METTL7A
Source: Front Cell Dev Biol. 2021 Nov 1;9:759192. doi: 10.3389/fcell.2021.759192 (PMC8591228; doi:10.3389/fcell.2021.759192)
Supplement: Supplementary file 3 [file Table_3.DOCX]

**Supplementary Table 3. Protein-mass spectrometry results**

| Accession | Name | Species | Peptides(95%) | |
| --- | --- | --- | --- | --- |
| NP_001304051 | Argonaute RISC component 1 | HUMAN | | 6 |
| NP_000537 | Cullin-7 | HUMAN | | 6 |
| NP_054733 | U5 small nuclear ribonucleoprotein 200 kDa helicase | HUMAN | | 5 |
| NP_005392 | protein tyrosine phosphatase non-receptor type 14 | HUMAN | | 5 |
| NP_005103 | WD repeat domain 1 | HUMAN | | 5 |
| NP_112740 | heterogeneous nuclear ribonucleoprotein D like | HUMAN | | 5 |
| NP_001014431 | Rapamycin-insensitive companion of mTOR | HUMAN | | 4 |
| NP_077739 | Desmocollin-1 | HUMAN | | 4 |
| NP_057030 | Protein pelota homolog | HUMAN | | 4 |
| NP_057856 | E3 ubiquitin-protein ligase BRE1A | HUMAN | | 3 |
| NP_001365343 | Histone deacetylase 4 | HUMAN | | 3 |
