# Supplementary figures and images for: miR-6807-5p Inhibited the Odontogenic Differentiation of Human Dental Pulp Stem Cells Through Directly Targeting METTL7A
Source: Front Cell Dev Biol. 2021 Nov 1;9:759192. doi: 10.3389/fcell.2021.759192 (PMC8591228; doi:10.3389/fcell.2021.759192)

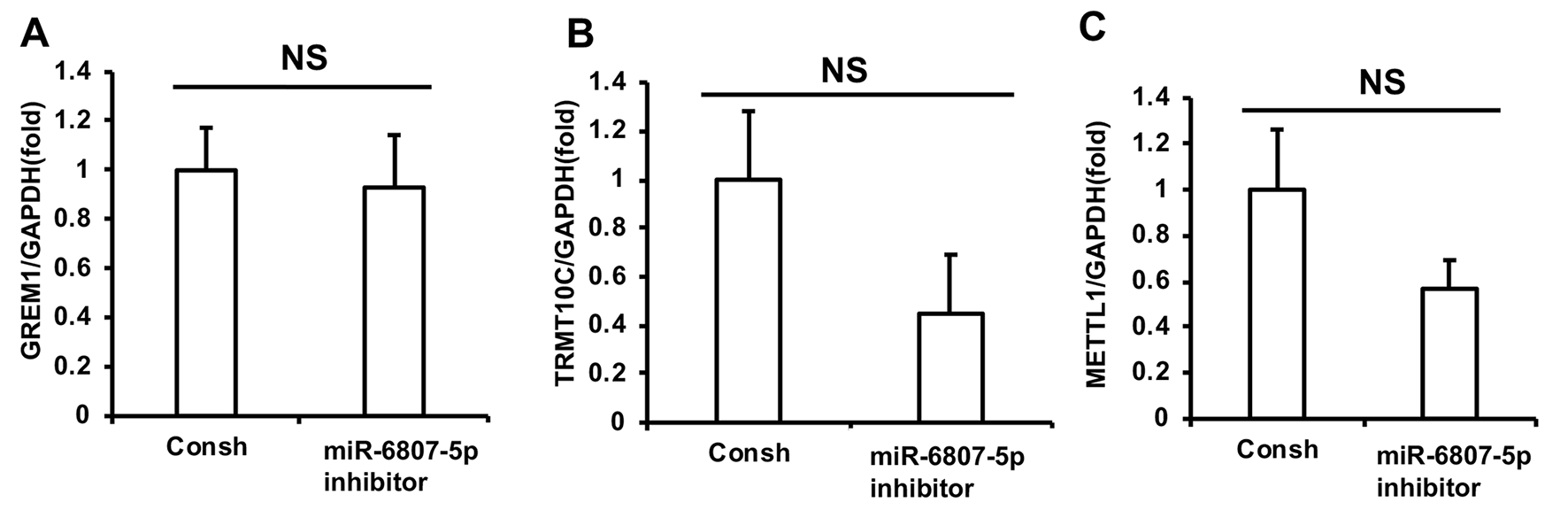

Supplement: Supplementary Figure 1 — The gene expressions were detected by real-time RT-PCR. (A–C) There was no significant difference of the expression of GREM1 (A), TRMT10C (B), and METTL1 (C) in the miR-6807-5p inhibitor group compared with the control group. GAPDH was used as internal control. Student’s t-test was performed to determine statistical significance. All error bars represent the standard deviation (n = 3). [file Image_1.TIF]
